# Low ACADM expression predicts poor prognosis and suppressive tumor microenvironment in clear cell renal cell carcinoma

**DOI:** 10.1038/s41598-024-59746-5

**Published:** 2024-04-25

**Authors:** Libin Zhou, Min Yin, Fei Guo, Zefeng Yu, Guobin Weng, Huimin Long

**Affiliations:** 1 Department of Urology, The Affiliated Lihuili Hospital, Ningbo University, Ningbo, China; 2 Departments of Urology, Ningbo Medical Center Lihuili Hospital, Ningbo, Zhejiang China; 3 Ningbo Institute for Medicine and Biomedical Engineering Combined Innovation, The Affiliated Lihuili Hospital, Ningbo University, Ningbo, China; 4 School of Information Engineering, Nanchang University, Nanchang, China; 5 Department of Urology, Ningbo Yinzhou No.2 Hospital, Ningbo, China

**Keywords:** Cancer, Tumour biomarkers, Tumour-suppressor proteins, Urological cancer, Computational biology and bioinformatics, Data mining, Gene ontology, Genetics, Gene expression, Genetic markers

## Abstract

Clear cell renal cell carcinoma (ccRCC) represents a highly frequent renal cancer subtype. However, medium-chain acyl-CoA dehydrogenase (ACADM) encodes an important enzyme responsible for fatty acid β-oxidation (FAO) and its association with prognosis and immunity in cancers has rarely been reported. Therefore, the present work focused on exploring ACADM’s expression and role among ccRCC cases. We used multiple public databases and showed the hypo levels of ACADM protein and mRNA within ccRCC. Additionally, we found that ACADM down-regulation showed a remarkable relation to the advanced stage, high histological grade, as well as dismal prognostic outcome. As suggested by Kaplan–Meier curve analysis, cases showing low ACADM levels displayed shorter overall survival (OS) as well as disease-free survival (DFS). Moreover, according to univariate/multivariate Cox regression, ACADM-mRNA independently predicted the prognosis of ccRCC. In addition, this work conducted immunohistochemistry for validating ACADM protein expression and its prognostic role in ccRCC samples. KEGG and GO analyses revealed significantly enriched genes related to ACADM expression during fatty acid metabolism. The low-ACADM group with more regulatory T-cell infiltration showed higher expression of immune negative regulation genes and higher TIDE scores, which might contribute to poor response to immunotherapies. In conclusion, our results confirmed that downregulated ACADM predicted a poor prognosis for ccRCC and a poor response to immunotherapy. Our results provide important data for developing immunotherapy for ccRCC.

## Introduction

Renal cell carcinoma (RCC) is among the ten most frequent cancers, which occupies ≤ 3% of adult cancers^1^. Among these, 16% displayed distant metastatic disease and exhibited a 5-year survival rate of only 11.6%^2^. Clear cell renal cell carcinoma (ccRCC) shows the highest morbidity among RCC pathological subtypes^3^. The ccRCC is a metabolic disease, generally accompanied by the reprogramming of metabolisms, including glucose and lipid metabolism^4,5^. Several cancer studies have shown that the changes in metabolic pathways control tumor energetics and biosynthesis^6^. Notably, changes of fatty acid metabolism during carcinogenesis have been more and more explored for the functions in sustaining growth, satisfying energy demands, and offering metabolites in anabolism^7,8^. Different from the lipogenic phenotype, the function of mitochondrial fatty acid β-oxidation (FAO) has not been well defined in cancer.

Current literature could not confirm whether it was the upregulation or downregulation of FAO that contributed to tumorigenesis, which was attributed to the nature of tumor heterogeneity. Numerous malignancies reported the overexpression of FAO enzymes, which were responsible for the proliferation, survival, stemness, drug resistance, or metastasis. Blocking FAO could attenuate tumor growth in several tumor models^9^. However, mitochondrial content in ccRCC showed inverse relation to tumor grade, suggesting that suppressing mitochondrial activity might be critical for ccRCC development^10^. Furthermore, many enzymes responsible for FAO had reduced expression within high-grade tissues, indicating that acyl-CoAs were not oxidized within the RCC tissue^11^. These findings highlighted that the downregulation of FAO was related to the tumorigenesis of ccRCC.

The first step in catalyzing FAO in mitochondria involves medium-chain acyl-CoA dehydrogenase (ACADM) degrading medium-chain fatty acids. Medium-chain acyl-CoA dehydrogenase deficiency results from ACADM mutations, which represents a frequently seen hereditary metabolic diseases among the Caucasian population^12^. This suggests that ACADM significantly affected metabolic disorders. Patients experiencing cardiovascular, nonalcoholic and metabolic fatty liver diseases have shown alteration in ACADM expression^13–16^. To the best of our knowledge, ACADM knockdown enhances hepatocellular carcinoma (HCC) proliferation^17^. Similarly, neuroblastoma patients with high ACADM expressions exhibit better overall survival (OS); the upregulation of ACADM and FAO by the tozasertib can suppress neuroblastoma progression^18^. Nonetheless, ACADM remains unexplored in terms of its prognostic value as well as associated mechanism in ccRCC.

In this study, we used electronic databases, clinical samples and cells to determine ACADM expression levels. We also analyzed relation of ACADM with ccRCC patient prognosis. Next, this work explored the functional enrichment of ACADM, immune cell infiltration, and response to immunotherapy. Our findings demonstrated that ccRCC patients with downregulated ACADM levels displayed a poor prognosis and poor response to immunotherapy due to the infiltration of immunosuppressive cells.

## Materials and methods

### Data source

This work obtained the clinical and mRNA information of kidney renal clear cell carcinoma (KIRC) in The Cancer Genome Atlas (TCGA). Next, we used the GEO database to obtain more than 443 specimens, including GSE15641^19^, GSE36895^20^, GSE46699^21^, GSE53000^22^, and GSE53757^23^. Simultaneously, we selected another dataset, including 91 RCC and 45 non-carcinoma samples from the ICGC database.

### Gene expression and survival analysis

This work utilized TIMER2.0 online tool for exploring differential ACADM-mRNA expression in non-carcinoma tissues compared with cancer samples in pan-cancers^24^. Next, the CPTAC (Clinical proteomic tumor analysis consortium) model in the UALCAN portal (http://ualcan.path.uab.edu/index.html) was used to evaluate the total ACADM protein among 12 types of cancers^25,26^. The immunofluorescence staining images based on the Human Protein Atlas (HPA, www.proteinatlas. org) showed ACADM protein subcellular localization in A-431 and U251 cells^27^. The “Survival Map” and “Survival Analysis” modules in the GEPIA2.0 database (http://gepia2.cancer-pku.cn/#analysis) were used to examine the association among the median expression of ACADM-mRNA and the OS and disease-free survival (DFS) rate in pan-cancer and single-cancer types^28^.

### Methylation analysis

The UALCAN online tool was applied to determine ACADM methylation in ccRCC^25^. We loaded the methylation 450 data of KIRC from the UCSC Xena (https://xenabrowser.net/) and used it to research the CpG sites in the ACADM promoter. Furthermore, we used the SMART online tool (http://www.bioinfo-zs.com/smartapp/) to analyze the association of the number of CpG sites between the ACADM promoter and ACADM-mRNA, along with determining the different levels of CpG sites between ccRCC and normal samples^29^.

### Cell culture

We purchased five cell lines (HK-2, ACHN, 786-O, 769-P, and Caki-1) from Procell Life Science &Technology Co., China and cultivated them within MEM or RPMI 1640 or McCoy’s 5A medium (Procell, Wuhan, China) with 10% fetal bovine serum (FBS) under a 5% 5% CO_2_ environment and 37 °C.

### Real-time quantitative PCR (qPCR)

The present work adopted Trizol reagent for isolating total RNA. Next, RT-PCR Master Mix (TOYOBO, Japan) was used to reverse transcribe RNA into cDNA, which was further analyzed through qPCR by an SYBR Premix ExTaq kit (TOYOBO, Japan). The ACADM forward primer was 5ʹ-GGAAGCAGATACCCCAGGAAT-3ʹ and reverse primer was 5ʹ-AGCTCCGTCACCAATTAAAACAT-3ʹ. The results were normalized using GAPDH, and the relative mRNA expression was calculated using the 2 − ΔΔCT method.

### Western-bloting (WB) Assay

This work conducted WB assay in line with our previous study^30^. For this, we electrophoresed proteins (50 µg) onto SDS-PAGE gel, followed by transfer onto PVDF membranes. Next, membranes were incubated with the ACADM (1:2000, Abcam, Britain, ab92461) or GAPDH (1:2000, Abcam, Britain, ab8245) antibodies and visualized them using ECL (Coolaber, Beijing, China), with GAPDH being the endogenous control.

### Patients and specimens

Tissue chips (HkidE180su02), including 150 ccRCC and 30 tumor-adjacent tissues, were provided by Shanghai Outdo Biotech Company. The patient surgery was conducted between February 2008 and March 2010, and the follow-up period was extended till August 2015, i.e., from 5.5 to 7.5 years. All the included patient samples had complete clinical characteristics and follow-up information. This study gained approval from the Ethics Committee of Shanghai Outdo Biotech Company (Ethics number: SHYJSCP-1510001). Informed consent was obtained from all participants.

### Immunohistochemistry (IHC)

The immunostaining method was carried out in line with prior description^30^. The tissue chip was subjected to overnight incubation using ACADM antibody (1:5000, Abcam, Britain, ab92461) under 4 °C. Staining intensitie were categorized into 0–3, indicating no, weak, moderate and strong staining, separately, while stained cancer cell percentage was rated as 1–4, indicting 0–25%, 25–50%, 50–75%, and ≥ 75%, separately. The eventual score was determined by their product.

### Protein–protein interaction analysis

This study applied STRING database for analyzing potential binding proteins of ACADM^31^, while the parameters were set as follows: evidence was set as the meaning of network edges, experiments as active interaction sources, median confidence as the minimum required interaction score, and 250 as the maximum number of interactors.

### Functional enrichment analysis

The functional annotations of the intersected genes were determined using the Gene Ontology (GO) together with Kyoto Encyclopedia of Genes and Genomes (KEGG) analysis through clusterProfiler package, followed by visualization using ggplot2 package^32^.

### Immune infiltration analysis

We detected 22 immune cells within TCGA-KIRC samples using CIBERSORT algorithm and also explored the correlation among them and determined the differences in their levels^33^.

### TISCH2 analysis

Tumor Immune Single-cell Hub 2 (TISCH2) is a scRNA-seq database that focuses on the tumor microenvironment. It includes 190 databases and 6297320 cells from both tumor patients and healthy donors^34^. We used the “Dataset” model to determine the ACADM expression in different cells at the single-cell level.

### Immunotherapy sensitivity analysis

Based on ACADM expression, the Tumor Immune Dysfunction and Exclusion (TIDE) method was utilized for determining immunotherapy sensitivity in KIRC patients^35^.

### Statistical analysis

GraphPad Prism 7.0 and SPSS 23.0 were adopted for statistical analysis. Differences in continuous variables between two or multiple groups were calculated using Student’s t-test and ANOVA, respectively. Chi-square test was adopted to analyze differences in categorical variables. The Pearson’s or Spearman’s analysis was adopted for correlation analysis, while the impact of ACADM on survival and other clinical characteristics of ccRCC cases was identified by Cox regression and Kaplan–Meier analysis. P < 0.05 (two-sided) stood for statistical significance.

### Ethics approval

This study was performed in line with the principles of the Declaration of Helsinki. Approval was granted by The Ethics Committee of Shanghai Outdo Biotech Company (Ethics number: SHYJSCP-1510001).

## Results

### Expression and survival analysis of ACADM in pan-cancer

**Figure 1:**
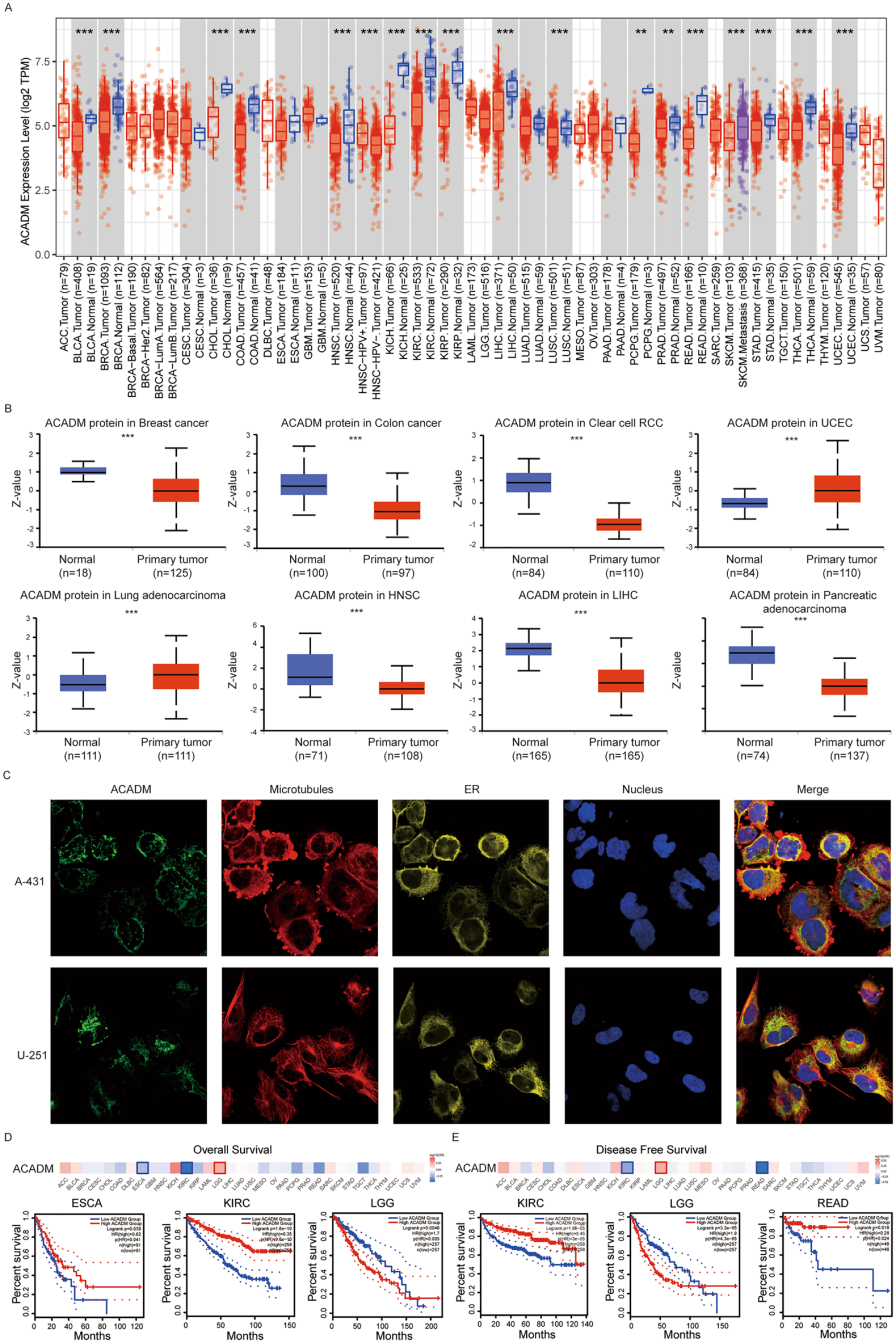
Expression and survival Analysis of ACADM in pan-cancer. (**A**) ACADM-mRNA in pan-cancer by TIMER2.0; (**B**) ACADM protein in pan-cancer by UALCAN; (**C**) The immunofluorescence images of ACADM protein, nucleus, endoplasmic reticulum (ER), microtubules and the incorporative images in A-431 and U251 cell lines derived from the HPA database; (**D)** The relationship between ACADM and overall survival in pan-cancer; (**E**) The relationship between ACADM and disease-free survival in pan-cancer. **P < 0.01, ***P < 0.001.

We employed the “Gene_DE” module of the TIMER2.0 web tool for exploring ACADM-mRNA expression pattern in pan-cancer. In comparison with adjacent non-carcinoma tissues, ACADM-mRNA expression decreased within BLCA (bladder urothelial carcinoma), BRCA (breast invasive carcinoma), CHOL (cholangiocarcinoma), COAD (colon adenocarcinoma), HNSC (head and neck squamous cell carcinoma), KICH (kidney chromophobe), KIRP (kidney renal papillary cell carcinoma), KIRC (kidney renal clear cell carcinoma), LIHC (liver hepatocellular carcinoma), LUSC (lung squamous cell carcinoma), READ (rectum adenocarcinoma), STAD (stomach adenocarcinoma), THCA (thyroid carcinoma), UCEC (uterine corpus endometrial carcinoma) (*P* < 0.001), PCPG (pheochromocytoma and paraganglioma), and PRAD (prostate adenocarcinoma) (*P* < 0.01, Fig. 1A). Furthermore, according to the CPTAC dataset, the ACADM protein was downregulated in colon cancer, breast cancer, HNSC, clear cell RCC, pancreatic adenocarcinoma, and hepatocellular carcinoma but upregulated in lung carcinoma and UCEC (Fig. 1B, P < 0.001). Based on the immunofluorescence results from the HPA database, ACADM protein showed major localization within mitochondria of the A-431 and U251 cells (Fig. 1C). Besides, relation of ACADM-mRNA with prognosis pan-cancer was analyzed, which suggested that ACADM down-regulation predicted dismal OS in ESCA (*P* = 0.039) and KIRC (*P* < 0.001, Fig. 1D). However, in KIRC (*P* < 0.001) and READ (*P* = 0.016), it was associated with poor DFS (Fig. 1E). Additionally, LGG (brain lower grade glioma) with high ACADM expression exhibited both poor OS (*P* = 0.005) and DFS (*P* < 0.001).

### Downregulation of ACADM within ccRCC

**Figure 2:**
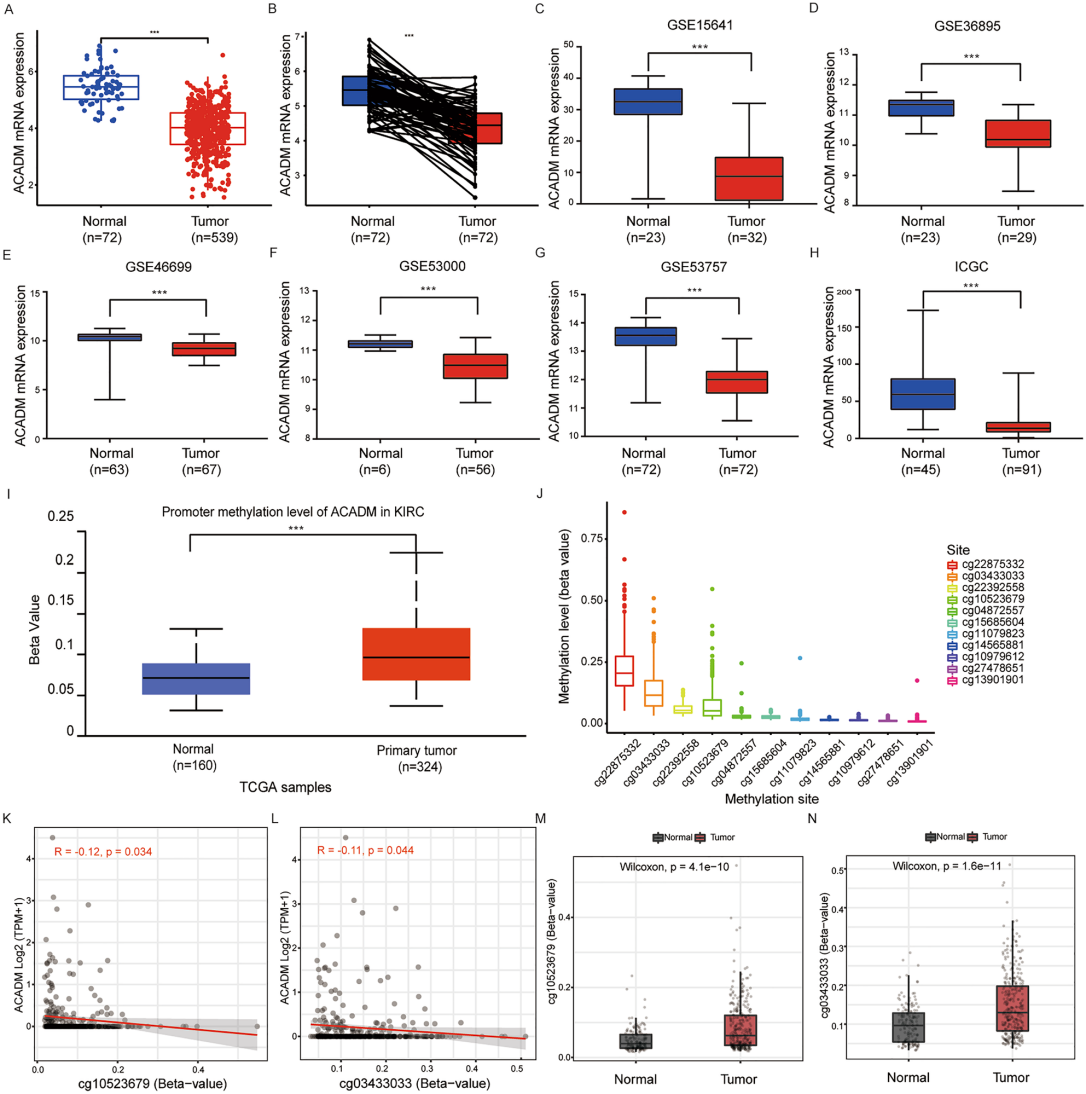
ACADM mRNA and methylation levels in ccRCC samples. (**A**) ACADM mRNA expression level 539 ccRCC samples and 72 adjacent normal samples from TCGA-KIRC database; (**B**) ACADM mRNA expression level in 72 paired ccRCC samples from TCGA-KIRC database; The different ACADM mRNA expression in GSE15641 (**C**), GSE36895 **(D)**, GSE46699 **(E)**, GSE53000 **(F)**, GSE53757 **(G)**, and ICGC **(H)**; (**I**) ACADM methylation levels between ccRCC and normal samples; (**J**) The types and levels of CpG sites in ACADM promoter; The correlation between ACADM mRNA and cg10523679 **(K)** and cg03433033 **(L)**. The different level of cg10523679 **(M)** and cg03433033 **(N)** between normal and tumor samples. ***P < 0.001.

Since we found a close association between ACADM and OS and DFS in ccRCC patients, we conducted further in-depth research on ccRCC. In the TCGA-KIRC database, ACADM-mRNA was found significantly downregulated in ccRCC compared to normal controls (Fig. 2A,B). Similar results were also observed in five GEO and one ICGC dataset (Fig. 2C–H). DNA methylation, a common form of epigenetic regulation, can silence gene expression. Hence, we analyzed the ACADM promoter methylation levels to explore the potential mechanism underlying decreased ACADM expression in ccRCC. The UALCAN database showed a higher methylation level of ACADM promoter in KIRC compared to normal samples (Fig. 2I). According to the methylation 450 data of KIRC obtained from the UCSC Xena, the levels of 11 CpG sites were analyzed (Fig. 2J). As shown in Fig. 2K,L, the SMART online tool showed a significant negative correlation between ACADM-mRNA of cg10523679 and cg03433033. Also, a significant difference was observed in the cg10523679 and cg03433033 levels between normal and tumor samples (Fig. 2M,N).

### Relationships between ACADM-mRNA and clinical factors among ccRCC patients

**Figure 3:**
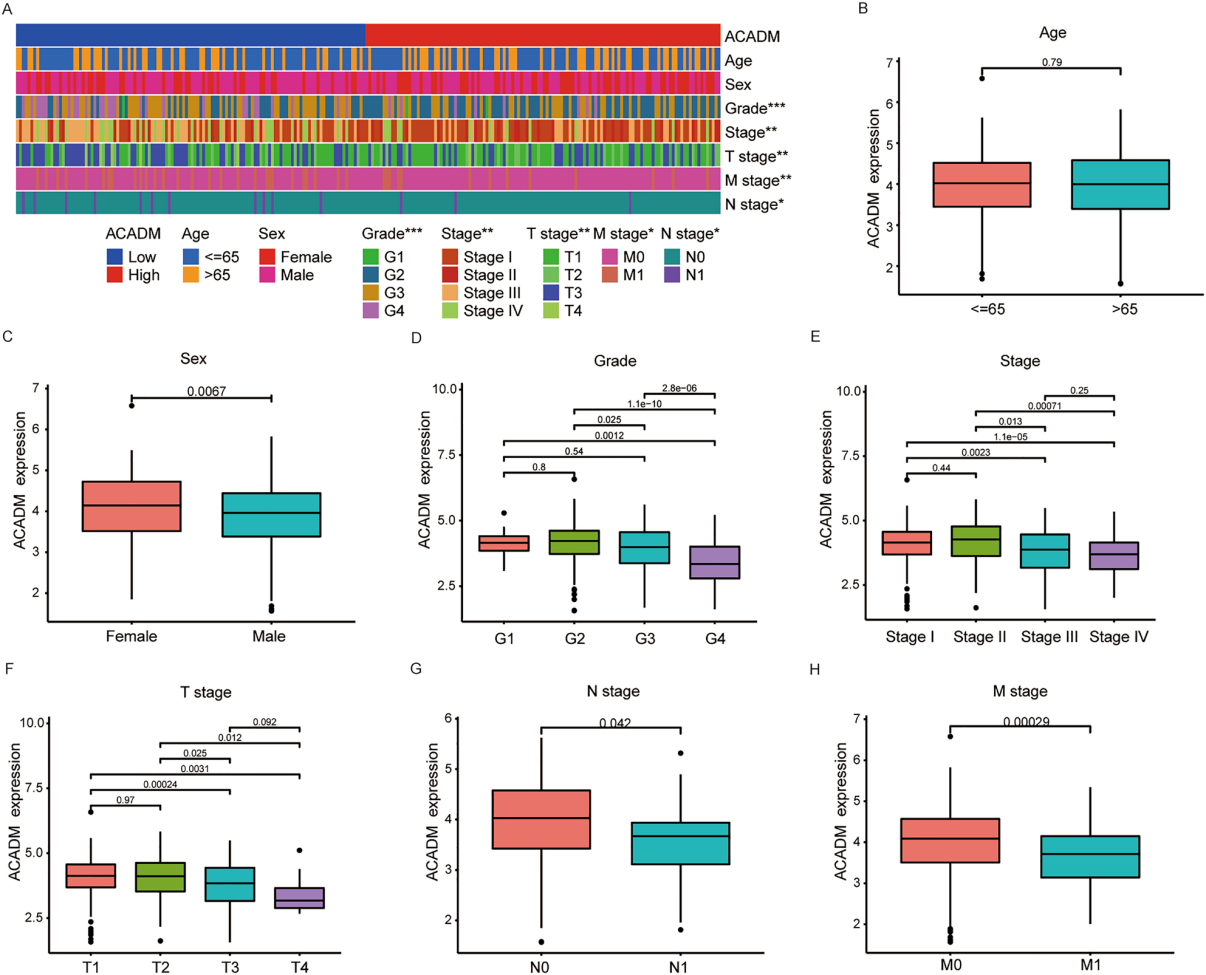
Relationships between ACADM expression and clinicopathological factors in ccRCC patients. (**A**) The heatmap of ACADM expression and clinicopathological factors; ACADM expression in different groups, age **(B)**, sex **(C)**, grade **(D)**, stage** (E)**, T stage **(F)**, N stage **(G)**, and M stage **(H)**. *P < 0.05, **P < 0.01, ***P < 0.001.

We used 246 ccRCC patients having complete clinical data in TCGA-KIRC database for exploring relation of ACADM-mRNA with clinical factors. The ACADM-mRNA levels showed a significant association with the grade (*P* < 0.001), stage (*P* < 0.001), T (*P* = 0.004), N (*P* = 0.05), M stages (*P* = 0.014) and vital status (*P* < 0.001) but not with age (*P* = 0.725) or sex (*P* = 0.085, Table S1, Fig. 3A). Spearman’s analysis suggested that the ACADM-mRNA levels were negatively correlated to the grade (*P* < 0.001), stage (*P* < 0.001), T stage (*P* < 0.001), N stage (*P* = 0.011), M stage (*P* = 0.008) and vital status (*P* < 0.001, Table S2). Furthermore, we observed a significant differential ACADM expression among different sex (Fig. 3C), grade (Fig. 3D), stage (Fig. 3E), T stage (Fig. 3F), N stage (Fig. 3G), and M stage (Fig. 3H). However, no significant difference was observed in the age group (*P* = 0.79, Fig. 3B).

### Prognostic value of ACADM-mRNA in ccRCC cases

**Figure 4:**
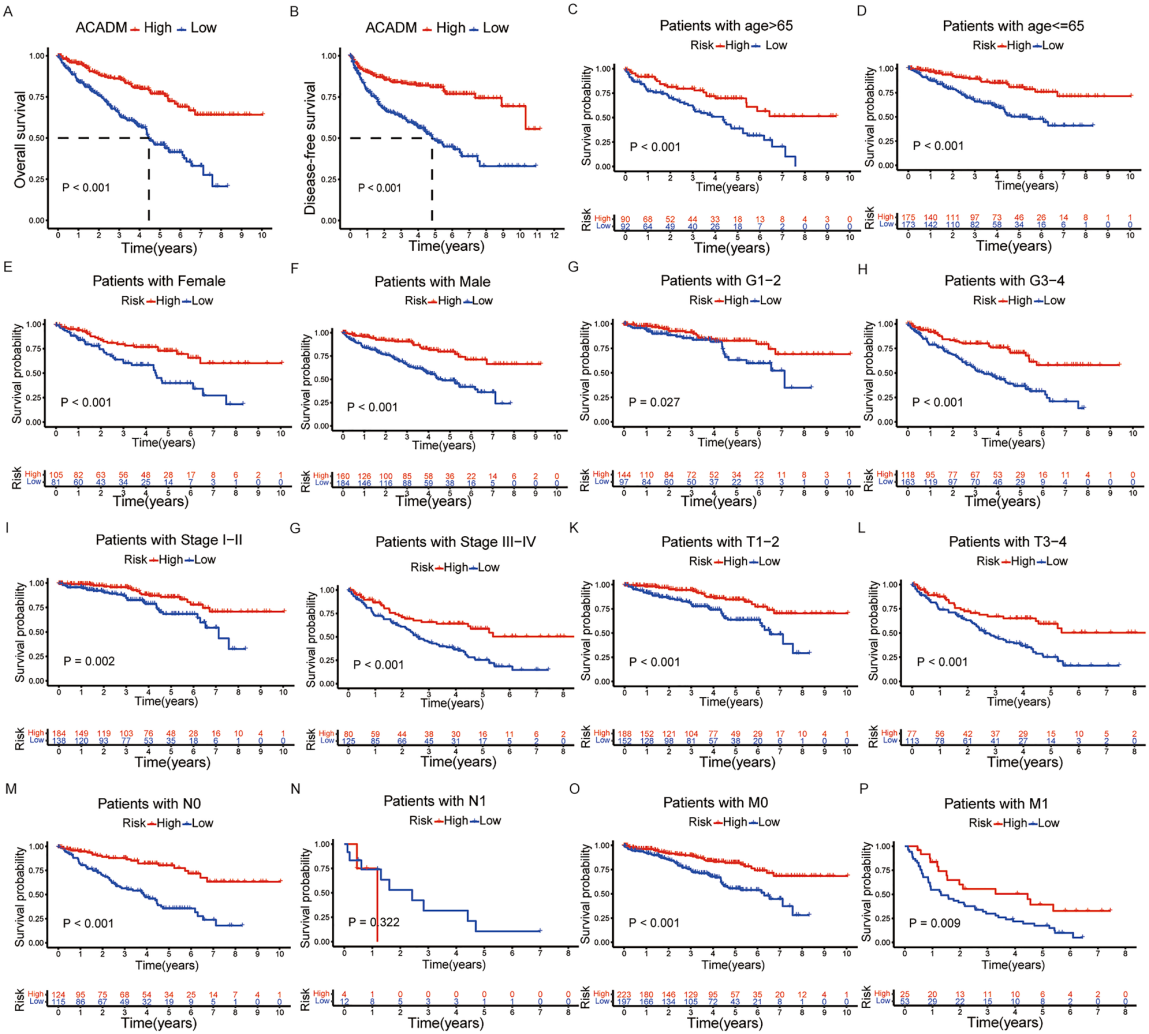
Survival analysis of ACADM mRNA in TCGA-KIRC patients. (**A**) Overall survival analysis; (**B**) Progression free survival analysis; (**C-P**) The OS stratified by the clinical subgroups.

For studying ACADM-mRNA expression’s value in predicting ccRCC prognosis, Kaplan–Meier curves and the TCGA-KIRC dataset were used. OS (Fig. 4A) and DFS (Fig. 4B) of ccRCC cases showing ACADM down-regulation markedly shortened compared with those showing up-regulation (*P* < 0.001). Besides, differences were significant between up- and down-regulation groups in OS rate among the clinical subgroups, except for N1 (Fig. 4C–P).

**Figure 5:**
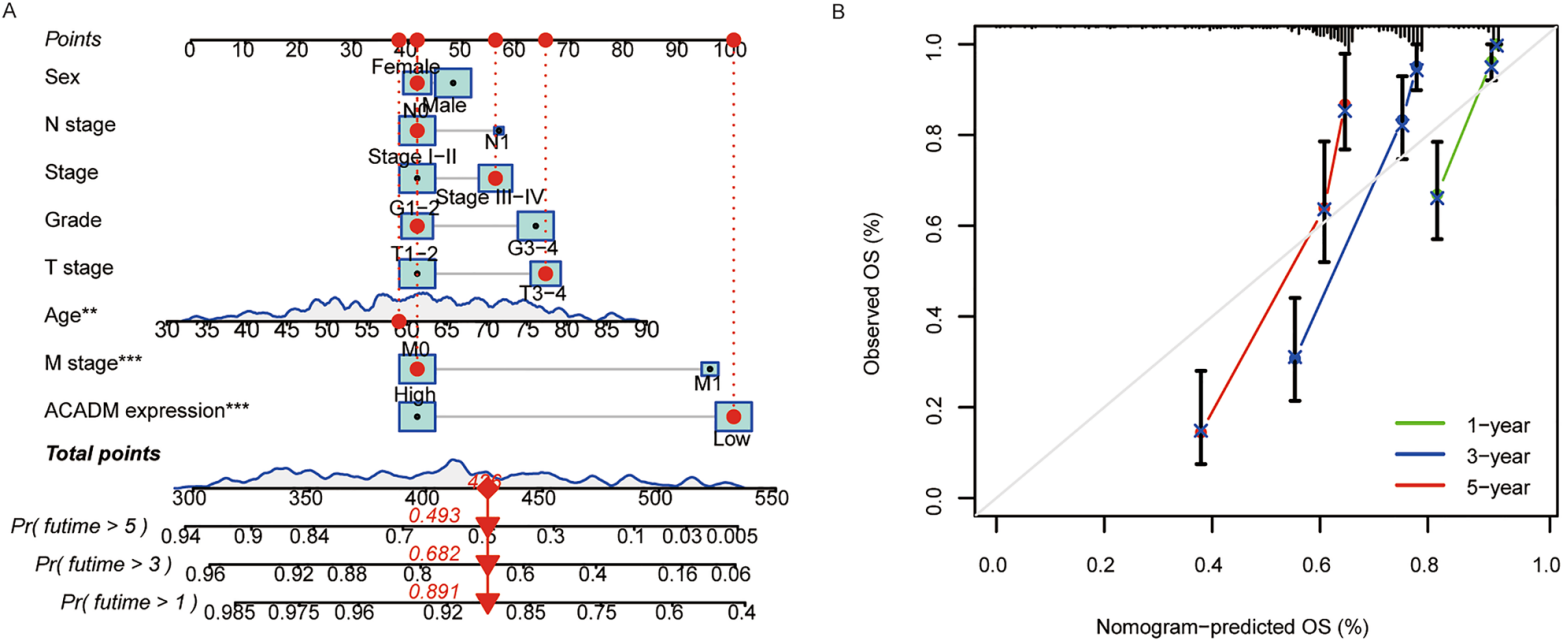
The nomogram and calibration plots. (**A)** A nomogram to predict the 1-, 3-, and 5-year overall survival probability of ccRCC patients; (**B)** A calibration plot of the nomogram. **P < 0.01, ***P < 0.001.

Furthermore, univariate/multivariate Cox analysis was conducted for determining if ACADM-mRNA independently predicted TCGA-KIRC prognosis. The univariate analysis showed that low ACADM-mRNA expression significantly predicted dismal OS and DFS (HR 0.508; 95%CI 0.406–0.634, *P* < 0.001, Table S3), multivariate analysis suggested that ACADM (HR 0.550; 95%CI 0.428–0.706, *P* < 0.001) independently predicted OS and DFS of ccRCC cases (Table S3). Finally, the TCGA-KIRC database was used to establish a nomogram plot and a calibration plot, which predicted the OS probability in ccRCC patients (Fig. 5). Overall, these results implied that ACADM-mRNA independently predicted ccRCC prognosis.

### ACADM protein expression within RCC cell and tissues

**Figure 6:**
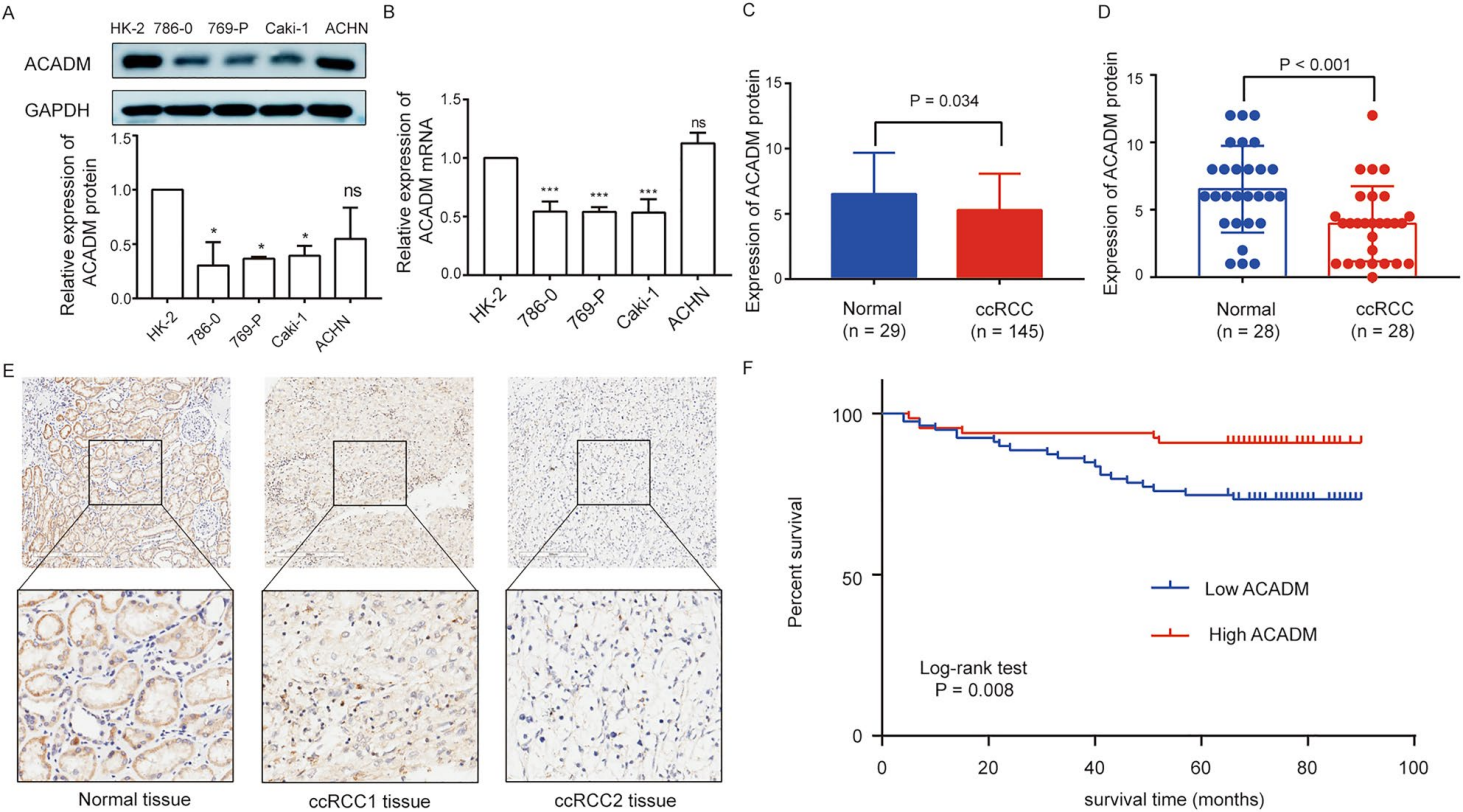
The ACADM expression in RCC cells and ccRCC tissues. (**A**) The ACADM protein expression in RCC cells (the original blots/gels were presented in Supplementary Fig. 1); (**B**) ACADM-mRNA expression in RCC cell lines; (**C)** ACADM protein expression between 29 normal and 145 ccRCC tissues; (**D)** ACADM protein expression in 28 paired ccRCC and para-carcinoma samples; (**E**) The representative IHC staining images of ACADM protein; (**F**) Kaplan–Meier survival curves for overall survival of ACADM. **P < 0.01, ***P < 0.001, ns means no significance.

We examined ACADM protein expression within cells and clinical specimens. Relative to healthy kidney cells, ACADM protein (Fig. 6A) and mRNA (Fig. 6B) showed lower expression in RCC cell lines. Furthermore, the IHC staining of 150 ccRCC and 30 para-carcinoma specimens was performed and scored using a standard method. However, five ccRCC and one para-carcinoma tissues were off target. Compared to the 29 normal controls, ACADM protein was significantly downregulated in 145 ccRCC samples (*P* < 0.05, Fig. 6C). Also, it was significantly downregulated in 28 paired ccRCC and para-carcinoma samples (*P* < 0.05, Fig. 6D). Representative IHC images are displayed in Fig. 6E. Based on our findings, ACADM protein level decreased within ccRCC tissues.

Next, we investigated relation of ACADM protein with clinical features within ccRCC by clustering 145 ccRCC samples into ACADM up- and down-regulation groups based on mean ICH scores. Detailed clinical characteristics are shown in Table 1. According to our results, ACADM protein expression was markedly related to sex (*P* = 0.048), grade (*P* = 0.036), stage (*P* = 0.027), T stage (*P* = 0.039), and vital status (*P* = 0.007) but not to age (*P* = 0.126) and N stage (*P* = 0.457, Table 1). Furthermore, Spearman’s analysis revealed that ACADM protein levels were markedly negatively related to grade (*P* = 0.031), stage (*P* = 0.012), T stage (*P* = 0.009), and vital status (*P* = 0.009, Table S4). As revealed by Kaplan–Meier analysis, cases showing ACADM down-regulation exhibited poorer OS (Fig. 6F, P = 0.008). Subsequently, the univariate analysis showed that ACADM down-regulation predicted OS in ccRCC cases (HR 0.315; 95% CI 0.127–0.781, *P* = 0.013, Table S5). However, based on multivariate regression, ACADM protein did not independently predicted OS of ccRCC patients (Table S5).

**Table 1:** Association between ACADM protein and clinical characteristics of ccRCC patients in clinical samples.

Characteristic	No.of cases (%)	ACADM expression		*P*-value
		Low	High	
Age				
< 65	110 (75.9)	56	54	0.126
≥ 65	35 (24.1)	23	12	
Sex				
Female	41 (28.3)	17	24	0.048
Male	104 (71.7)	62	42	
Grade				
G1	21 (14.5)	10	11	0.036
G2	94 (64.8)	46	48	
G3	26 (17.9)	19	7	
G4	4 (2.8)	4	0	
Stage				
Stage I	119 (82.1)	59	60	0.027
Stage II	13 (9.0)	11	2	
Stage III	12 (8.3)	9	3	
Stage IV	1 (0.7)	0	1	
T stage				
T1	119 (82.1)	59	60	0.039
T2	14(9.7)	11	3	
T3	12 (8.3)	9	3	
N stage				
N0	142 (97.9)	78	64	0.457
N1	3 (2.1)	1	2	
Vital status				
Alive	118 (81.4)	58	60	0.007
Dead	27 (18.6)	21	6	

### Functional enrichment of ACADM

**Figure 7:**
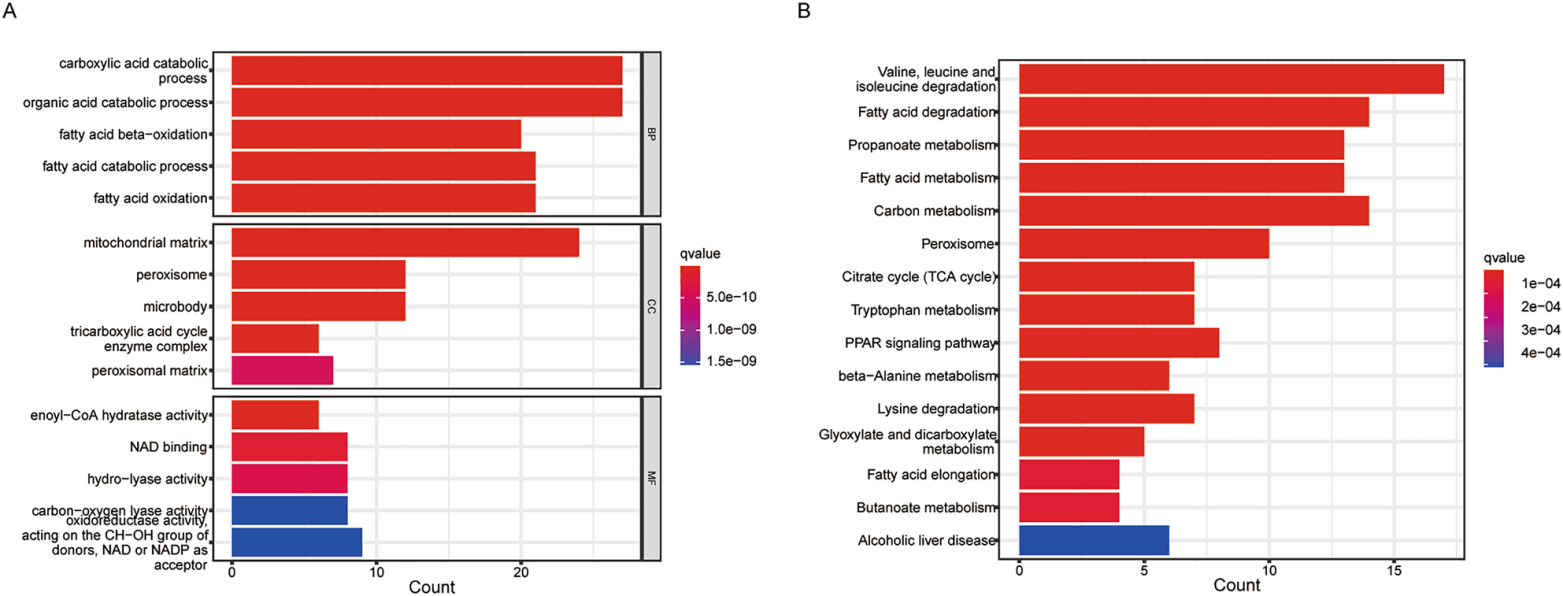
Functional enrichment of ACADM. (**A)** GO analysis of ACADM-related genes; (**B)** KEGG analysis of ACADM-related genes.

For investigating ACADM-related mechanism underlying cancer occurrence, ACADM-binding proteins and their correlated genes were screened for functional analysis. Consequently, the STRING database was used to screen a total of 217 ACADM-binding proteins while 1389 correlated genes was screened out according to the |correlation coefficient |> 0.5 and *P* < 0.05. Overall, 54 intersected genes were obtained (Table S6) and subjected to GO and KEGG analysis. According to GO, genes in the biological progress (BP) were enriched into the carboxylic acid catabolic process, fatty acid beta-oxidation, fatty acid catabolic process, fatty acid oxidation, enoyl-CoA hydratase activity, and so on (Fig. 7A). These genes provided cellular components (CC) in the mitochondrial matrix, peroxisome, and microbody, with an important role in the molecular function (MF) of enoyl-CoA hydratase activity, NAD binding, and hydrolase activity (Fig. 7A). KEGG pathway analysis indicated enrichment in the degradation of valine, leucine, isoleucine, and fatty acids, as well as propanoate metabolism, fatty acid metabolism, and so on (Fig. 7B).

### Correlation of ACADM with immune infiltration in ccRCC

**Figure 8:**
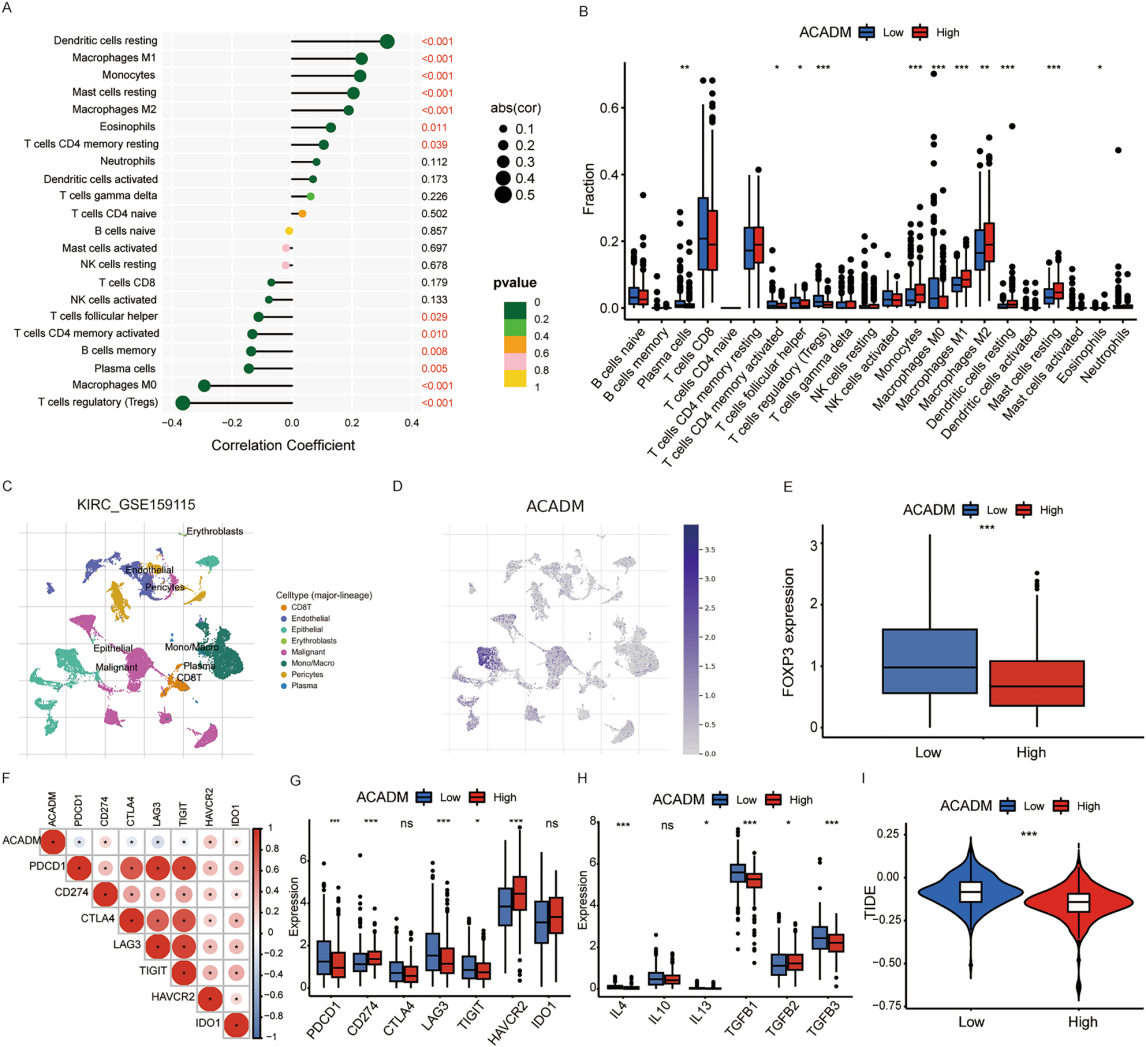
Correlation of ACADM with immune cells. (**A**) The correlation between ACADM expression and immune cells; (**B**) The different infiltration of immune cells between high- and low-ACADM expression groups; (**C**) The cell types in KIRC_GSE159115 dataset; (**D**) Distribution of ACADM in different cells in KIRC_GSE159115 dataset; (**E**) FOXP3 expression in the high- and low-ACADM groups; (**F**) Correlation between the risk score and common immune checkpoints; (**G**) Expression levels of the common immune checkpoints between the high- and low-ACADM groups; (**H**) Expression of the cytokines between the high- and low-ACADM groups; (**I**) The difference of TIDE score between high- and low-ACADM expression groups. *P < 0.05, **P < 0.01, ***P < 0.001.

To explore whether ACADM influenced immune cell infiltration, we used the CIBERSORT method. Spearman’s correlation analysis showed a negative relationship between ACADM expression and regulatory T cells (r =  − 0.364, *P* < 0.001), macrophages M0 (r =  − 0.292, *P* < 0.001), plasma cells (r =  − 0.144, *P* = 0.005), memory B cells (r =  − 0.136, *P* = 0.008), activated CD4 memory T cells (r =  − 0.132, *P* = 0.010), follicular helper T cells (r =  − 0.112, *P* = 0.029) but a positive relationship with resting dendritic cells (r = 0.317, *P* < 0.001), macrophages M1 (r = 0.232, *P* < 0.001), monocytes (r = 0.227, *P* < 0.001), macrophages M2 (r = 0.189, *P* < 0.001), eosinophils (r = 0.130, *P* = 0.011), and resting CD4 memory T cells (r = 0.106, *P* = 0.039, Fig. 8A). Various analyses suggested a significant increase of monocytes, macrophages M1, macrophages M2, resting dendritic cells, resting mast cells, and eosinophils in high-ACADM group, and a significant increase of plasma cells, activated CD4 memory T cells, follicular helper T cells, regulatory T cells (Tregs), and macrophages M0 in low-ACADM group (Fig. 8B). Furthermore, the KIRC_GSE159115 dataset from the TISCH online database was used to evaluate ACADM expression at the single-cell level. The results showed the malignant cells exhibited low ACADM expression when compared to the epithelial cells, which was consistent with the IHC results (Fig. 8C,D). Compared to other immune cells, mono/macro cells showed higher ACADM expression. In accordance with the Treg infiltration, FOXP3 (factor forkhead box protein P3, marker for Tregs) was more highly expressed in low-ACADM group than in high-ACADM group (Fig. 8E). These data indicated that the patients in low-ACADM group presented an immunosuppressive phenotype due to the infiltration of Treg cells. To confirm the immunosuppressive phenotype, common immune checkpoints and cytokines were further evaluated. The correlation analysis found that the ACADM expression had negative relationships with PDCD1, CTLA-4, LAG-3, and TIGIT, and positive relationships with CD274, HAVCR2 and IDO1 (Fig. 8F). The histogram also indicated that the expression levels of PDCD1, LAG-3, and TIGIT in low-ACADM group were significantly higher than those in high-ACADM group (Fig. 8G). Cytokines (TGF-β, IL-4, and IL-10) involved in the immunosuppressive process were also significantly upregulated in low-ACADM group except TGF-β2 (Fig. 8H). Finally, the TIDE method was used to evaluate ACADM’s effect on estimating immunotherapy response. Compared to high-ACADM group, low-ACADM group showed higher TIDE scores, which implied that the patients in low-ACADM group had poor efficacy to the immunotherapy (*P* < 0.001, Fig. 8I). In summary, these results suggested that ACADM expression influenced immune cell infiltration and predicted the response to immunotherapy in ccRCC patients.

## Discussion

The characteristic manifestations of ccRCC include increased cholesterol ester storage and adipogenesis with clear cytosol. The adipogenesis-related protein activity in ccRCC patients has demonstrated abnormalities in lipid metabolism^36,37^. FAO, similar to the additional extensively studied metabolic pathways, is also related to cancer. It exhibits dysregulation within different human cancers and is related to different cancer occurrence aspects, including growth, survival, metastasis, stemness, and drug resistance^38^. Numerous studies in cancer patients have shown decreased enzyme involvement in FAO^39,40^. Therefore, the upregulation of FAO may suppress tumor cell growth while arresting their cell cycle^41,42^. ACADM is upregulated during FAO, which breaks down fatty acids into acetyl-CoA in the mitochondria, thereby participating in the citric acid cycle^43^. Several studies have revealed the important value of ACADM in the prognosis of different cancers. For example, high expression of ACADM in GBM was shown to impair mitochondrial function and glioblastoma growth in vitro and in vivo^44^. Also, in HCC with decreased ACADM expression, the modulation of β-oxidation resulted in enhanced cell aggressiveness^45^. However, the ACADM expression and function in ccRCC remain unknown.

In this study, we observed decreased levels of ACADM-mRNA and protein in most types of tumors, which revealed that ACADM might serve as a tumor suppressor gene, which was probably involved in tumor development. Furthermore, based on immunofluorescent staining, ACADM protein showed major location within mitochondrion of A-431 and U-251 cells, signifying its functional association with the β-oxidation of mitochondria^12^. Next, GEPIA2.0 database was adopted for analyzing relationship of ACADM level with prognosis pan-cancer and found that while poor OS and DFS were shown by KIRC patients with low ACADM expression, LGG patients with high ACADM expression also showed poor OS and DFS, indicating the different mechanisms between KIRC and LGG. However, little research has been conducted on the function and mechanism of ACADM in KIRC. Hence, we focused on KIRC for further study.

The TGCA-KIRC and GEO datasets revealed downregulated ACADM-mRNA levels in ccRCC samples. According to our study, decreased ACADM might be attributed to DNA hypermethylation. Moreover, downregulated ACADM-mRNA was related to poor clinicopathological features, including higher grade, advanced stage, higher T stage, and distant metastasis. Based on univariate/multivariate Cox regression, ACADM-mRNA independently predicted OS and DFS. Consistently, our stratified analysis based on different clinical characteristics also confirmed that cases showing ACADM down-regulation displayed remarkably dismal OS compared with those showing ACADM up-regulation, indicating that ACADM downregulation predicted dismal prognostic outcome. The nomogram, including age, M stage, and ACADM expression, signified an excellent clinical application value in the estimation of ccRCC survival. Finally, we used cancer cell lines and clinical tissues to confirm the bioinformatics results of ACADM. Compared to HK-2 cells, both ACADM mRNA and protein expression was downregulated within RCC cell lines. Furthermore, immunohistochemical staining showed decreased ACADM protein levels in ccRCC samples. The Chi-square test showed that ACADM protein was strongly correlated to sex, grade, stage, T stage, and vital status, while Spearman’s analysis revealed that the ACADM protein showed negative relation to the grade, stage, and T stage. Both univariate/multivariate Cox regression implied that although ACADM level could predicted OS among ccRCC cases, it did not serve as an independent factor.

Additionally, bioinformatic analyses were performed for exploring ACADM’s bioactivity in modulating ccRCC. According to GO and KEGG analyses based on the related genes, ACADM showed strong relation to fatty acid metabolism, such as fatty acid β-oxidation as well as fatty acid degradation. CcRCC is aggressive cancer arising from the proximal tubular epithelium^6^. Single-cell analysis showed that epithelial cells displayed high expression of ACADM. This indicated that normal kidney epithelium, while developing into ccRCC, underwent adipogenic transdifferentiation due to the downregulation of ACADM. Lipid metabolism contributes to metabolic reprogramming and unbridled cell growth in ccRCC^46^. Interestingly, a prior work reported that elevating HIF expression by reducing FAO added a new layer of benefit to ccRCC tumors^47^, where the decreased expression of ACADM might have delayed β-oxidation causing fatty acid accumulation, which induced fatty acid metabolic reprogramming and tumor deterioration^6^.

Renal cell carcinoma is one of the most immune-infiltrated tumors^48^. Since the infiltration of immune cells within tumor microenvironment has an important effect on the regulation of cancer development, tumor immune cell percentage within cancer may have also influenced the disease biology, the prognosis and the response to immunotherapy in ccRCC patients^49,50^. Moreover, FAO can be reprogrammed within cancer-associated immune as well as additional host cells, thus facilitating immunosuppression and tumor-promoting microenvironment^38^. So, we used CIBERSORT method to analyze immune cell infiltration between high- and low-ACADM groups according to TCGA-KIRC data. The results showed that Tregs showed negative relation to ACADM and were at higher levels in low-ACADM group than high-ACADM group. Tregs characterized by the expression of the master transcription factor FOXP3 suppress anticancer immunity, thereby hindering protective immunosurveillance of tumors and hampering effective antitumor immune responses in tumor-bearing hosts, constitute a current research hotspot in the field^51^. Elevated Treg cells within the tumor microenvironment have been observed in ccRCC patients and correlated with disease progression and poor prognosis^52^. In our study, the FOXP3 expression and Tregs were high in low-ACADM group, implying that ACADM might influence the Tregs infiltration in the tumor microenvironment and the prognosis of ccRCC patients. Tregs can suppress immune activation by secreting immune-suppressive cytokines (IL-10, IL-35, and TGF-β) or expressing coinhibitory molecules such as CTLA-4, PD-1, LAG-3, and TIGIT^53^. Cytokines (IL-4, IL-10, IL-13, TGF-β1, and TGF-β3) and checkpoints (PDCD1, LAG-3, and TIGIT) involved in immune suppression were highly expressed in the low-ACADM group, which were attributed to the infiltration of Tregs. We further studied the relationship between the ACADM expression and the response to immunotherapy by the TIDE algorithm. Notably, the TIDE score in the low-ACADM group was higher than that in the high-ACADM group, which indicating an undesirable immunotherapy response because of the greater amounts of Tregs. These results imply that the expression level of ACADM has the potential to predict infiltrating immune cells in ccRCC, which might be beneficial for the immunotherapy.

Although our research has revealed the ACADM expression level and its potential role in immune infiltration and prognosis of KIRC, the work has several limitations. First, the proportions of immune cells in KIRC were mainly based on online data. Second, further intensive analysis on ACADM-related biological mechanisms is required in the future, which needs rigorous wet lab experiments.

To sum up, the present work demonstrates ACADM’s role in predicting ccRCC prognosis, suppressive immune microenvironment, and immunotherapy sensitivity. Our findings may offer some vital clues for the development of novel therapies for ccRCC.

### Supplementary Information

Supplementary Information 1.

Supplementary Information 2.

Supplementary Information 3.

Supplementary Information 4.

Supplementary Information 5.

Supplementary Information 6.

Supplementary Information 7.

## Data Availability

The datasets analyzed during the current study are available in the TCGA database (https://portal.gdc.cancer.gov), GEO databases (https://www.ncbi.nlm.nih.gov/geo/query/acc.cgi?acc=GSE15641, https://www.ncbi.nlm.nih.gov/geo/query/acc.cgi?acc=GSE36895, https://www.ncbi.nlm.nih.gov/geo/query/acc.cgi?acc=GSE46699, https://www.ncbi.nlm.nih.gov/geo/query/acc.cgi?acc=GSE53000, https://www.ncbi.nlm.nih.gov/geo/query/acc.cgi?acc=GSE53757) and ICGC database (https://dcc.icgc.org/). The code and related datasets used in the overall bioinformatic analyses are available on the Github (https://github.com/zlburo/ACADM_2023). All additional information generated during and/or analysed during the current study are available from the corresponding author on reasonable request.
