# Supplementary material for: Low ACADM expression predicts poor prognosis and suppressive tumor microenvironment in clear cell renal cell carcinoma
Source: Sci Rep. 2024 Apr 25;14:9533. doi: 10.1038/s41598-024-59746-5 (PMC11045743; doi:10.1038/s41598-024-59746-5)
Supplement: Supplementary file 2 — Supplementary Information 2. [file 41598_2024_59746_MOESM2_ESM.pdf]

Low ACADM expression predicts poor prognosis and suppressive tumor microenvironment in clear cell renal cell carcinoma

Libin Zhou, Min Yin, Fei Guo , Zefeng Yu, Guobin Weng & Huimin Long

Table S1 Association between ACADM expression and clinical characteristics of ccRCC patients in TCGA-KIRC

Characteristic	No.of cases (%)	ACADM expression		*P*-value
		Low	High	
Age				
<65	144 (58.5)	74	70	0.503
⩾65	102 (41.5)	48	54	
Sex				
Female	97 (39.4)	41	56	0.064
Male	149 (60.6)	81	68	
Grade				
G1	3 (1.2)	1	2	<0.001
G2	106 (43.1)	38	68	
G3	100 (40.7)	54	46	
G4	37 (15.0)	29	8	
Stage				
Stage I	101 (41.1)	38	63	0.001
Stage II	32 (13.0)	12	20	
Stage III	71 (28.9)	43	28	
Stage IV	42 (17.1)	29	13	
T stage				
T1	105 (42.7)	42	63	0.005
T2	40 (16.3)	17	23	
T3	94 (38.2)	57	37	
T4	7 (2.8)	6	1	
N stage				
N0	232 (94.3)	111	121	0.026
N1	14 (5.7)	11	3	
M stage				
M0	205 (83.3)	94	111	0.009
M1	41 (16.7)	28	13	
Vital status				
Alive	154 (62.6)	54	100	<0.001
Dead	92 (37.4)	68	24	
