# Supplementary material for: Low ACADM expression predicts poor prognosis and suppressive tumor microenvironment in clear cell renal cell carcinoma
Source: Sci Rep. 2024 Apr 25;14:9533. doi: 10.1038/s41598-024-59746-5 (PMC11045743; doi:10.1038/s41598-024-59746-5)
Supplement: Supplementary file 3 — Supplementary Information 3. [file 41598_2024_59746_MOESM3_ESM.pdf]

Low ACADM expression predicts poor prognosis and suppressive tumor microenvironment in clear cell renal cell carcinoma

Libin Zhou, Min Yin, Fei Guo , Zefeng Yu, Guobin Weng ＆ Huimin Long

Table S2 Correlation of ACADM mRNA expression with clinical characteristics

Characteristics	ACADM expression	
	Spearman correlation	*P*-value
Age	0.049	0.442
Sex	-0.105	0.101
Grade	-0.315	<0.001
Stage	-0.272	<0.001
T stage	-0.247	<0.001
N stage	-0.162	0.011
M stage	-0.17	0.008
Vital status	-0.388	<0.001
