# Supplementary material for: Low ACADM expression predicts poor prognosis and suppressive tumor microenvironment in clear cell renal cell carcinoma
Source: Sci Rep. 2024 Apr 25;14:9533. doi: 10.1038/s41598-024-59746-5 (PMC11045743; doi:10.1038/s41598-024-59746-5)
Supplement: Supplementary file 4 — Supplementary Information 4. [file 41598_2024_59746_MOESM4_ESM.pdf]

Low ACADM expression predicts poor prognosis and suppressive tumor microenvironment in clear cell renal cell carcinoma

Libin Zhou, Min Yin, Fei Guo , Zefeng Yu, Guobin Weng ＆ Huimin Long

Table S3 Univariate and multivariate survival analysis of clinical characteristics and ACADM expression with OS and DFS in ccRCC patients

Characteristics	Univariate analysis						Multivariate analysis					
	OS			DFS			OS			DFS		
	HR	95%CI	*P*-value	HR	95%CI	*P*-value	HR	95%CI	*P*-value	HR	95%CI	*P*-value
ACADM	0.508	0.406-0.634	<0.001	0.527	0.421-0.660	<0.001	0.550	0.429-0.706	<0.001	0.5881	0.452-0.765	<0.001
Age	1.023	1.005-1.041	0.012	0.996	0.978-1.013	0.623	1.030	1.011-1.050	0.002	1.016	0.995-1.037	0.136
Sex	1.013	0.666-1.541	0.951	1.163	0.750-1.804	0.500	1.164	0.747-1.814	0.501	1.472	0.896-2.417	0.127
Grade	2.242	1.682-2.988	<0.001	2.440	1.801-3.306	<0.001	1.247	0.898-1.733	0.188	1.237	0.875-1.748	0.229
Stage	1.862	1.541-2.251	<0.001	2.910	2.298-3.685	<0.001	1.252	0.744-2.105	0.398	2.245	1.350-3.734	0.002
T stage	1.943	1.538-2.456	<0.001	2.397	1.868-3.076	<0.001	1.046	0.643-1.702	0.856	0.873	0.585-1.300	0.503
N stage	2.932	1.516-5.668	0.001	3.777	1.940-7.353	<0.001	1.408	0.685-2.893	0.352	1.208	0.571-2.556	0.621
M stage	4.073	2.634-6.300	<0.001	8.795	5.616-13.775	<0.001	2.475	1.090-5.617	0.030	2.321	1.007-5.347	0.048

OS overall survival, DFS disease-free survival, HR hazard ratio, CI confidence interval
