# Supplementary material for: Low ACADM expression predicts poor prognosis and suppressive tumor microenvironment in clear cell renal cell carcinoma
Source: Sci Rep. 2024 Apr 25;14:9533. doi: 10.1038/s41598-024-59746-5 (PMC11045743; doi:10.1038/s41598-024-59746-5)
Supplement: Supplementary file 5 — Supplementary Information 5. [file 41598_2024_59746_MOESM5_ESM.pdf]

Low ACADM expression predicts poor prognosis and suppressive tumor microenvironment in clear cell renal cell carcinoma

Libin Zhou, Min Yin, Fei Guo , Zefeng Yu, Guobin Weng ＆ Huimin Long

Table S4 Correlation of ACADM protein with clinical characteristics

Variables	ACADM expression	
	Spearman correlation	*P*-value
Age	-0.089	0.287
Sex	-0.102	0.224
Grade	-0.179	0.031
Stage	-0.209	0.012
T stage	-0.217	0.009
N stage	0.028	0.738
Vital status	-0.216	0.009
