# Supplementary material for: Low ACADM expression predicts poor prognosis and suppressive tumor microenvironment in clear cell renal cell carcinoma
Source: Sci Rep. 2024 Apr 25;14:9533. doi: 10.1038/s41598-024-59746-5 (PMC11045743; doi:10.1038/s41598-024-59746-5)
Supplement: Supplementary file 6 — Supplementary Information 6. [file 41598_2024_59746_MOESM6_ESM.pdf]

Low ACADM expression predicts poor prognosis and suppressive tumor microenvironment in clear cell renal cell carcinoma

Libin Zhou, Min Yin, Fei Guo , Zefeng Yu, Guobin Weng ＆ Huimin Long

Table S5 Univariate and multivariate survival analysis of clinical characteristics and ACADM protein with OS in ccRCC clinical samples

Characteristcs	Univariate analysis			Multivariate analysis		
	HR	95% CI	*P*-value	HR	95% CI	*P*-value
ACADM	0.315	0.127-0.781	0.013	0.431	0.158-1.178	0.101
Age	1.046	1.010-1.084	0.012	1.039	0.998-1.082	0.065
Sex	2.429	0.840-7.025	0.101			
Grade	1.29	0.748-2.224	0.36			
Stage	4.079	2.783-5.979	<0.001	5.665	1.134-28.313	0.035
T stage	4.327	2.862-6.541	<0.001	0.59	0.109-3.180	0.539
N stage	22.666	6.067-84.686	<0.001	2.463	0.491-12.362	0.273

OS overall survival, HR hazard ratio, CI confidence interval
