# Supplementary material for: Low ACADM expression predicts poor prognosis and suppressive tumor microenvironment in clear cell renal cell carcinoma
Source: Sci Rep. 2024 Apr 25;14:9533. doi: 10.1038/s41598-024-59746-5 (PMC11045743; doi:10.1038/s41598-024-59746-5)
Supplement: Supplementary file 7 — Supplementary Information 7. [file 41598_2024_59746_MOESM7_ESM.pdf]

Low ACADM expression predicts poor prognosis and suppressive tumor microenvironment in clear cell renal cell carcinoma

Libin Zhou, Min Yin, Fei Guo , Zefeng Yu, Guobin Weng ＆ Huimin Long

Table S6 The intersected genes

DLD
SIRT1
ACO2
EHHADH
CAT
IREB2
BBOX1
ACAD11
HIBADH
CPT1A
ACAT1
SLC27A2
UQCRC2
BCKDHA
MUT
ACAA2
ACOX1
ADH5
CRYL1
PRDX3
IDH3A
BCKDHB
HADHB
ALDH9A1
HIBCH
PEX3
ACADSB
DBT
ABCD3
PGM2
SDHD
PCCA
AUH
TFAM
SDHB
HSD17B4
SCP2
CMPK1
ECHDC1

ALDH6A1
HSDL2
ECI2
NDUFS1
ACSL1
SUCLG2
ETFDH
HADHA
SUCLA2
CPT2
CHD9
NCOA2
HADH
PPARA
ETFA
